# Moderation Effect of Physical Activity on the Relationship Between Fear of COVID-19 and General Distress: A Pilot Case Study in Arabic Countries

**DOI:** 10.3389/fpsyg.2020.570085

**Published:** 2020-09-23

**Authors:** Tareq A. Alsalhe, Sulaiman O. Aljaloud, Nasr Chalghaf, Noomen Guelmami, Dallal W. Alhazza, Fairouz Azaiez, Nicola Luigi Bragazzi

**Affiliations:** ^1^College of Sport Sciences and Physical Activity, King Saud University, Riyadh, Saudi Arabia; ^2^Group for the Study of Development and Social Environment (GEDES), Faculty of Human and Social Science of Sfax, Sfax, Tunisia; ^3^Higher Institute of Sport and Physical Education of Sfax, University of Sfax, Sfax, Tunisia; ^4^Department of Neurosciences, Rehabilitation, Ophthalmology, Genetics, Maternal and Child Health (DINOGMI), Genoa University, Genoa, Italy; ^5^Laboratory for Industrial and Applied Mathematics (LIAM), Department of Mathematics, York University, Toronto, ON, Canada

**Keywords:** COVID-19, fear, gender, general distress, physical activity, partial least square modeling

## Abstract

**Aims:**

This study aimed to investigate the effects of the fear of COVID-19, level of physical activity, and gender on negative stress (distress) in an Arab population by means of structural equations based on partial least squares.

**Materials and Methods:**

The sample population comprised of 459 participants from four Arab countries (age *M* = 33.02, SD = 8.46; *n* = 237 women and *n* = 222 men). The level of education was basic (<9 years of study; *n* = 144), secondary/vocational (between 9 and 12; *n* = 178), and university (*n* = 137). The “Fear of COVID-19” Scale, the short form of the “International Physical Activity Questionnaire,” and the “Perceived Stress Scale” questionnaires were disseminated by emails and social networks *via* Google Forms. SMARTPLS software version 3.2.9 was used to model the relationships between the variables under study.

**Results:**

Results confirmed the links between level of physical activity, fear of COVID-19, and gender, showing a significant mediating effect of the fear of COVID-19 on the relationship between gender and general distress. The level of physical activity was also found to influence the fear of COVID-19, varying depending on gender. In addition, the model highlighted the presence of a moderation effect of the level of physical activity.

**Conclusion:**

Based on the model presented in the present study, we can conclude that the COVID-19 pandemic has a profound impact on psychological distress in the target populations. The impact of the level of physical activity on psychological distress is shown to be very important during the pandemic phase.

## Introduction

“Severe Acute Respiratory Syndrome Coronavirus type 2” (SARS-CoV-2) is the infectious agent responsible for the “Coronavirus disease 2019” (COVID-19), which represents an emerging communicable disorder characterized by an extremely high infection rate and a relatively high mortality. This viral outbreak that originated in the city of Wuhan, province of Hubei, mainland China, has been officially designated as a global pandemic by the World Health Organization (WHO), affecting the majority of countries around the world (Cucinotta and Vanelli, 2020; Rothan and Byrareddy, 2020). Great fear and perceived threat of the disease have been directly associated with its rate and route of transmission (spreading quickly and invisibly) as well as with its morbidity and mortality worldwide.

The consequences of the spread of the pandemic, its associated fear, and the impact of the repeated consumption of negative messages related to COVID-19 (Holmes et al., 2020), in addition to fake and “post-truth” news divulged on social networks, have affected not only vulnerable groups but also entire populations. Lessons learnt from previous natural disasters and hazards have demonstrated psychological effects to last longer than the episode itself, with a dramatically high economic impact. For this reason, all scientists are seriously concerned by both the physical and the mental health of individuals (Zhou et al., 2020).

In such a context, indeed many people in several countries are expected to experience emotional, cognitive, physical, and behavioral reactions. According to several studies, stress, anxiety, depression (Tan B. Y. et al., 2020; Tan W. et al., 2020), post-traumatic stress disorder symptoms (Chew et al., 2020), insomnia (Tan B. Y. et al., 2020; Tan W. et al., 2020), and suicidal ideation (Hao et al., 2020) have been reported in response to the pandemic spread. For example, during the *Ebola* outbreak, fear-related behaviors increased the population’s rates of suffering and psychiatric symptoms, which contributed to the increase in indirect mortality (Shultz et al., 2016).

Even for virus-free households, COVID-19 can act as a major stressor, resulting in chronic anxiety and exposing individuals to economic–financial hardship. All these can be even amplified by the public health interventions (self-isolation, social/physical distancing, quarantine, and lockdown) that have been implemented and enforced to curb the pandemic spread. Such highly stringent and restrictive policies can exacerbate the feelings of social isolation and can impair and disrupt social relationships. Besides the stress generated by the disease itself, the strictures of the confinement are unprecedented and, being completely new to citizens, can raise concerns about how people could react at the individual and collective levels. In such a context, individuals are expected to experience high levels of stress: this indeed occurs when subjects feel that their resources are not enough to cope with a given event or situation in a particularly demanding environment.

Stress levels may increase (Pfefferbaum and North, 2020; Polizzi et al., 2020; Qiu et al., 2020; Sun et al., 2020; Thakur and Jain, 2020; Wang et al., 2020a,b) both because of direct causes, such as fear of contracting the infection and anxiety or depression after being exposed to the virus (Rajkumar, 2020), and because of the consequences of the societal and economic–financial impact of the pandemic (Pfefferbaum and North, 2020; Wind et al., 2020). In addition, several families may be affected by school closing and lack of free school meals and may experience problems with employment and changes in daily habits. Wang et al. (2020a, b) assessed the emotional and the psychosocial effects of COVID-19 in 194 Chinese cities. The results of the study showed that approximately 16.5, 28.8, and 8.1% of the interviewees exhibited moderate-to-severe depression, anxiety, and stress, respectively. Gender (being female) and poor health status were statistically significant determinants of the psychological impact of the outbreak. In another Chinese survey conducted during the pandemic, the results highlighted a relevant prevalence rate of depression, anxiety, and stress-related symptoms (Liu N. et al., 2020; Liu S. et al., 2020).

Concerning the measures that can be taken to counteract such a burden, there is an increasing body of scholarly evidence documenting the advantages of exercising or practicing physical activity in terms of health and psychological gains (Stults-Kolehmainen et al., 2014). Indeed regular physical activity can result in positive behavioral changes and in the adoption of a healthy lifestyle, as such enhancing mental health and fostering abilities and skills to successfully face stressful events (Long, 1983; Salmon, 2001).

Several studies have shown that physical activity is an effective way to reduce stress in adults. Stults-Kolehmainen et al. (2014) reviewed 55 studies in depth. The majority of studies have indicated that psychological stress examined through objective (i.e., life events) and subjective (i.e., distress) measures is linked to a decrease in the level of physical activity and an increase in the adoption of sedentary lifestyles (Burg et al., 2017).

Despite the well-known advantages conferred by physical activity (Weyerer and Kupfer, 1994), this is not regularly practiced in Arab countries. Few people achieve the minimum level of physical activity necessary to maintain a good health status. During the pandemic, despite the quarantine and the restriction of movement, many people, especially males, began to practice physical activity in order to strengthen their immune system, improve mental health, and reduce the negative psychological impact of the measures implemented (Jiménez-Pavón et al., 2020). Recent scientometric analysis found that the most common research topics include emergency and surgical care, viral pathogenesis, and global responses to the COVID-19 pandemic, but there is a lack of research on the benefits of physical activity during the outbreak (Tran et al., 2020a,b).

Therefore, the aim of this investigation was to explore the impact of fear of COVID-19, level of physical activity, and gender on negative stress (distress) experienced in an Arab population by a second-generation statistical method. Indeed second-generation statistical techniques, called structural equation modeling (SEM), are currently applied by social scientists to model the causal links between unobservable variables.

Basically, there exist two major approaches to SEM: one being covariance-based (CB-SEM) and one based on partial least squares (PLS-SEM; also known as PLS path modeling). The former is mainly utilized to confirm (or reject) theories, whereas the latter is mainly employed to devise new theoretical models and frameworks. In this paper, given the novelty of the topic explored, the second approach (PLS-SEM) will be used.

## Materials and Methods

### Ethics Statement

The protocol of this investigation was fully approved by the UNESCO Chair “Health Anthropology Biosphere and Healing Systems,” University of Genoa, Genoa (Italy), the College of Sport Sciences and Physical Activity, King Saud University, Riyadh (Saudi Arabia), the Higher Institute of Sport and Physical Education of Sfax, Sfax (Tunisia), and the Faculty of Letters and Human Sciences of Sfax, Sfax (Tunisia). The Ethical Committee of the University of Sfax, Sfax, Tunisia, approved the project.

The present study was conducted in accordance with the ethical principles of the 1964 Helsinki Declaration and its subsequent amendments.

### Psychometric Instruments

#### The “Fear of COVID-19 Scale”

The adapted Arabic version of the “Fear of COVID-19 Scale,” developed by Alyami et al. (2020), was employed to quantitatively assess the fear of COVID-19. Reliability and validity were inspected in a sample of 693 Saudi participants and confirmed the uni-dimensional construct of the tool. The internal consistency was deemed satisfactory (α = 0.88), with a sound concomitant validity as demonstrated by statistically significant positive correlations (*r* = 0.66) with the “Hospital Anxiety and Depression Scale.”

The initial scale was examined in a sample of 717 Iranian participants. After evaluation, using both the classical test theory and the Rasch model, the properties of the scale were judged satisfactory: internal consistency (α = 0.82) and test–retest reliability (intra-correlation coefficient = 0.72) were indeed acceptable (Ahorsu et al., 2020).

Good psychometric properties similar to the original instrument have been proven for a Turkish version (Haktanir et al., 2020), an Italian adaptation (Soraci et al., 2020), and a model built in the Bangla population (Sakib et al., 2020).

The Turkish version (Haktanir et al., 2020) revealed its measurement robustness and the one-dimensional nature of the tool in an investigation conducted by recruiting a sample of 1,304 participants, aged 18–64 years, in 75 cities. A variety of analyses included confirmatory factor analysis, Item Response Theory, assessment of convergent validity, and internal consistency (namely, Cronbach’s α, McDonald’s ω, Guttmann’s λ6, and composite reliability). Cronbach’s alpha of the Italian version (Soraci et al., 2020) was also satisfactory (0.871), and the instrument displayed high reliability. Finally, results of the confirmatory factor analysis of the Bangla version (Sakib et al., 2020) confirmed the uni-dimensional factor structure of the scale and a very good internal reliability.

#### The Short Form of the “International Physical Activity Questionnaire”

The physical activity level was assessed by means of the validated Arabic version of the short form of the “International Physical Activity Questionnaire” (IPAQ) (Al-Hazzaa, 2007).

This tool has established good psychometric properties in several populations (Macfarlane et al., 2007; Lee et al., 2011; Vasheghani-Farahani et al., 2011).

The nine-item IPAQ enables to record self-reported physical activity level in the last 7 days. Scholars can convert the responses into metabolic equivalent task minutes per week according to a well-validated scoring protocol: total minutes spent on vigorous activity, moderate-intensity activity, and walking can be multiplied by 8.0, 4.0, and 3.3, respectively, to obtain metabolic equivalent of task (MET) scores for the different physical activity levels. The MET scores can be summed up to obtain the overall physical activity level. Based on the scores, three categories are possible: small, moderate, and high. In this research, we utilized the classification consisting of three categories to judge the level of physical activity practiced.

#### The Arabic Version of the “Perceived Stress Scale”

The “Perceived Stress Scale” (PSS) developed by Cohen et al. (1983) was employed to quantitatively measure the level of perceived stress. In particular, the 10-item PSS enables to assess the global perceived stress experienced in the past 30 days using a five-point scale (0 = never, 1 = almost never, 2 = once in a while, 3 = often, 4 = very often).

The Arabic version of the PSS was previously assessed in terms of reliability and validity, with acceptable results. Cronbach’s alpha coefficient was computed at 0.80 for the overall instrument. The test–retest reliability yielded an intra-correlation coefficient of 0.90 (Almadi et al., 2012).

### Data Collection and Procedures

In the context of the current study, a non-probabilistic, convenient sampling approach was utilized, and potential participants were contacted by email by the researchers contributing to the study from four Arab countries (Saudi Arabia: *n* = 157, Algeria: *n* = 102, Tunisia: *n* = 117, and Libya: *n* = 83).

The total sample comprised of 459 participants (age *M* = 33.02, *SD* age = 8.46), 237 women and 222 men. The level of education was basic (<9 years of study; *n* = 144), secondary/vocational (between 9 and 12; *n* = 178), and university (*n* = 137). The main characteristics are reported in Tables 1, 2.

**TABLE 1:** Age and gender characteristics of the recruited participants.

	**Frequency**	**%**	**Age (Mean)**	**Age (SD)**
**Gender**				
Male	222	48.4	33.13	8.990
Female	237	51.6	32.92	7.955

**TABLE 2:** Main characteristics of the recruited participants broken down according to education level, intensity of physical activity, and country.

	**Frequency**	**%**
**Country**		
Saudi Arabia	102	22.2
Algeria	157	34.2
Tunisia	83	18.1
Libya	117	25.5
**Age**		
18–20 years	11	2.4
21–30 years	171	37.3
31–40 years	214	46.6
41–50 years	40	8.7
More than 50 years	23	5.0
**Education level**		
Primary school	144	31.4
Middle school	178	38.8
High school	137	29.8
**Physical activity intensity**		
Low intensity	179	39.0
Middle intensity	141	30.7
High intensity	139	30.3

The multiple-choice questionnaire was filled by the participants via Google Forms from May 4 to May 15, 2020. The survey was sent to the participants and shared as well on social media and network platforms (including Twitter and Facebook).

After being advised of the study objectives, the respondents gave their free and informed consent before starting the survey.

No financial incentives were provided to the participants, and anonymity was maintained to ensure the confidentiality and the reliability of data.

### Statistical Software and Statistical Analysis

Descriptive data analysis was conducted by means of the commercial software “Statistical Package for the Social Sciences” (IBM SPSS software for Windows, version 26.0, IBM Corp., Armonk, NY, United States; released 2012), while PLS path modeling was carried out using SmartPls Software 3.2.9 (Ringle et al., 2015).

Two steps were carried out sequentially. First, the measurement model was evaluated and then the structural model was assessed.

For all the statistical analyses performed, figures with *p*-value equal to or less than 0.05 were considered as statistically significant.

## Results

**FIGURE 1:**
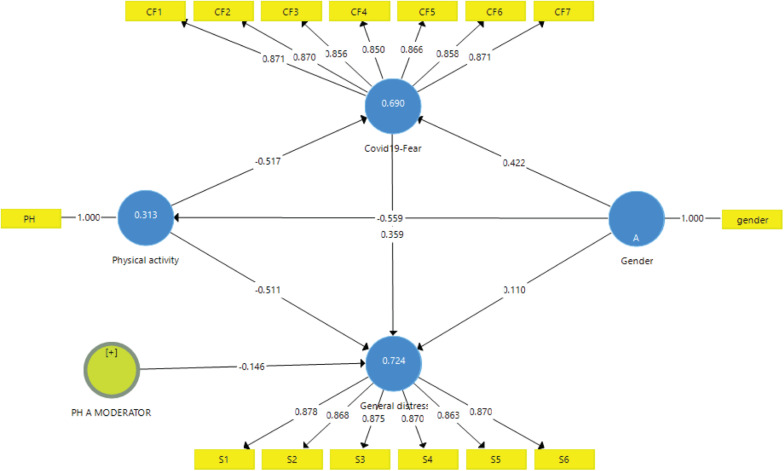
The main findings of the partial least squares algorithm reporting path coefficients and *R*^2^ values.

Figure 1 pictorially shows the findings of the PLS analysis, showing the path coefficients (β), the path statistical significance (*p*-value), and the variance explained by the structural model (in terms of *R*^2^ values).

### Assessment of the Measurement Model

The assessment of the measurement model is a fundamental step, providing useful details in terms of the reliability and the validity of the scales employed to assess latent constructs and their observed indicators (Loehlin, 1998).

There are different criteria that can be utilized to assess the reliability or the internal consistency of a psychometric instrument: (i) Cronbach’s alpha, which is based on the inter-correlations of the observed indicators (if greater than 0.70, reliability is acceptable; if greater than 0.80, it is considered excellent), (ii) composite reliability (Hair et al., 2016), and (iii) reliability coefficient of Dijkstra–Henseler’s rho (ρA) (Dijkstra and Henseler, 2015). The latter indices have a recommended cutoff value of 0.70 (Hair et al., 2016).

Table 3 shows the values of the three indices for the two measurement scales of COVID-19 and general distress which, being greater than 0.70, present adequate reliability.

**TABLE 3:** Internal consistency/reliability and average variance extracted.

**Constructs**	**Internal consistency/reliability**			**Average variance extracted**
	**Cronbach’s alpha**	**Rho_A**	**Composite reliability**	
COVID-19 fear	0.943	0.943	0.953	0.745
General distress	0.936	0.936	0.949	0.758

#### Convergent Validity

It shows the extent to which observable indicators can converge to form a latent construct representative of the data.

Convergent validity is measured using the mean extracted variance (AVE) which must be greater than 0.5. The results of our study show that the AVEs have acceptable values of 0.745 for COVID-19 fear and 0.758 for the general distress scale (see Table 3).

#### Discriminant Validity

Two criteria are widely used for assessing discriminant validity: Fornell–Larcker criterion [developed by Fornell and Larcker (1981)] and heterotrait–monotrait (HTMT) [proposed by Henseler et al. (2015)]. The Fornell–Larcker criterion specifies that the square root of the AVE of each construct should be greater than the construct’s highest correlation with any other construct in the model, while the HTMT is an estimate of the factor correlation (Table 4).

**TABLE 4:** The Fornell–Larcker criterion.

	**COVID-19 fear**	**Gender**	**General distress**	**Physical activity**
COVID-19 fear	0.863			
Gender	0.711	1.000		
General distress	0.775	0.629	0.871	
Physical activity	−0.753	−0.559	−0.792	1.000

To distinguish between two factors, the HTMT should be significantly less than 1. Henseler et al. (2015) suggested that values should be below 0.9 or, better, below 0.85.

Table 5 provides inference statistics for the HTMT values. These values should be smaller than 0.85 and demonstrate good reliability.

**TABLE 5:** The heterotrait–monotrait ratio of correlations.

	**Fear of COVID-19**	**Gender**	**General distress**
Gender	0.732		
General distress	0.824	0.650	
Physical activity	0.775	0.559	0.818

#### Indicator Reliability

Another way to assess an individual indicator’s reliability is to look at outer loadings for indicator constructs, which show how much variance is explained by the observed variable in terms of latent constructs (Hair et al., 2016). Table 6 reports the outer loadings of all indicators of constructs, which are greater than the minimum acceptable value (0.7).

**TABLE 6:** Indicator reliability.

	**COVID-19 fear**	**General distress**
CF1	0.871	
CF2	0.870	
CF3	0.856	
CF4	0.850	
CF5	0.866	
CF6	0.858	
CF7	0.871	
S1		0.878
S2		0.868
S3		0.875
S4		0.870
S5		0.863
S6		0.870

### Assessment of the Structural Model

A structural model can be employed to assess the linear regression effects of the endogenous construct upon one another (Hair et al., 2016) by specifying the pattern of the relationships among the various constructs (Loehlin, 1998).

#### Collinearity Assessment

To investigate the presence of collinearity within the structural model, tolerance or variance inflation factor (VIF) criteria can be applied (Hair et al., 2011). All the indicators of the constructs under study have a VIF value less than 5 (Table 7), indicating the absence of collinearity between the indicators.

**TABLE 7:** Collinearity assessment.

**Indicators**	**VIF**	**Indicators**	**VIF**
CF1	3.126	S1	3.214
CF2	3.144	S2	2.976
CF3	2.927	S3	3.198
CF4	2.859	S4	3.214
CF5	3.085	S5	3.135
CF6	2.859	S6	3.288
CF7	3.183		

#### Coefficient of Determination

According to previous work, the coefficient of determination (or *R*^2^ value) denotes the amount of variation in the dependent variable(s) that can be explained by one or more predictors, ranging between 0 and +1 and indicating the predictive accuracy of the structural model. According to Chin (1998), cutoff values of 0.19, 0.33, and 0.67 indicate a weak, moderate, and strong coefficient of determination, respectively.

Table 8 shows that fear of COVID-19, level of physical activity, and gender can together explain 72.4% of the variation of general distress in the population. In addition, gender and physical activity can explain 69.0% of the variance of fear of COVID-19, while 31.3% of the variance in physical activity can be explained by a gender effect.

**TABLE 8:** *R*^2^ of endogenous constructs.

**Scale**	***R*^2^**
COVID-19 fear	0.690
General distress	0.724
Physical activity	0.313

#### *f*^2^ Effect Size

According to Cohen (1988, p. 413), 0.02 *f*^2^ values for the significant independent variables indicate a weak effect, whereas 0.15 and 0.35 indicate moderate and substantial effects, respectively.

Table 9 shows that the effect sizes of COVID-19 fear, gender, and physical activity on general distress are 0.111, 0.030, and 0.333, respectively. This shows that physical practice has a medium effect size, while COVID-19 fear and gender have low effect sizes. In addition, great effects of gender and physical activity on fear of COVID-19 have been highlighted. Finally, a large size effect of physical activity on gender was recorded.

**TABLE 9:** Effect size (*f*^2^).

	**COVID-19 fear**	**General distress**	**Physical activity**
COVID-19 fear		0.111	
Gender	0.395	0.030	0.455
Physical activity	0.593	0.333	

#### Predictive Relevance *Q*^2^

Stone–Geisser’s *Q*^2^ value (Geisser, 1974; Stone, 1974) measure is an indicator of predictive power or predictive relevance. Running the blindfolding procedure, we got *Q*-values greater than zero (Table 10), indicating that our path model’s predictive relevance is high.

**TABLE 10:** Predictive relevance *Q*^2^.

	**Sum of squares of prediction errors (SSO)**	**Sum of squares of observations (SSE)**	***Q*^2^ (=1 − SSE/SSO)**
COVID-19 fear	3,213.000	1,574.892	0.510
General distress	2,754.000	1,279.696	0.535
Physical activity	459.000	316.647	0.310

##### Standardized root mean square residual

According to Henseler and Sarstedt (2013), the standardized root mean square residual (SRMR), computed as the difference between the observed correlation and the predicted correlation, can be considered as an absolute goodness-of-fit measure particularly adequate for PLS-SEM-based models. Values less than 0.10 up to 0.08 (in a conservative sense) are judged as a good fit (Hair et al., 2016). Our model has a good SRMR (0.034).

### Effects Testing

To validate the direct and the indirect link hypotheses, a bootstrapping procedure (Davison and Hinkley, 1997) is generally used to test the statistical significance of the coefficients.

Tables 11, 12 report both significant direct and indirect effects in the path model and show means, standard deviation, and *t*- and *p*-values.

**TABLE 11:** Results of bootstrapping for structural model direct effects evaluation.

	***M***	**SD**	***T***	***P*-values**
COVID-19 fear → general distress	0.361	0.051	7.001	0.000
Gender → COVID-19 fear	0.421	0.037	11.304	0.000
Gender → general distress	0.108	0.047	2.365	0.018
Gender → physical activity	–0.559	0.035	16.091	0.000
Physical activity (moderator) → general distress	–0.147	0.036	4.086	0.000
Physical activity → COVID-19 fear	–0.519	0.038	13.656	0.000
Physical activity → General distress	–0.511	0.047	10.778	0.000

**TABLE 12:** Results of bootstrapping for structural model indirect effects evaluation.

**Paths**	***M***	**SD**	***T***	***P*-values**
Gender → physical activity → COVID-19 fear	0.290	0.028	10.233	0.000
Gender → COVID-19 fear → general distress	0.152	0.026	5.794	0.000
Physical activity → COVID-19 fear → general distress	–0.187	0.030	6.225	0.000
Gender → physical activity → COVID-19 Fear → general distress	0.105	0.018	5.762	0.000
Gender → physical activity → general distress	0.286	0.033	8.774	0.000
Physical activity (moderator) → general distress	–0.15	0.03	4.54	0.000

Both the cause-and-effect relationships imply that exogenous constructs directly affect endogenous ones without any systematic influence from other variables. However, the inclusion of a third variable in the analysis may impact on the model’s relationships. The two major effects are mediation and moderation.

Mediation occurs when a third variable (called the mediating variable) is present in the model (example, COVID-19 fear). A change in the exogenous construction (for example, gender) may lead to a change in the mediating variable, which, in its turn, modifies the endogenous construction (for example, general distress).

In the present research, we have a mediating effect of COVID-19 fear which intervenes in the relationship between gender and general distress. At this level, a gender effect (the highest score attributed to the female gender) increases the fear of COVID-19 and therefore the general distress. In addition, another mediating effect is given by the level of physical activity: in the presence of this variable, the fear of COVID-19 varies by gender.

The other effect present in the model is moderation: when it is present, the direction and the strength of a given relationship between two constructions depend on a third variable. As such, the nature of the relationship between COVID-19 fear and general distress can differ depending on the values of the third variable (in our case, the level of physical activity). In other words, people who engage in physical activity and are afraid of COVID-19 do not have a great deal of stress compared to those who do not.

**FIGURE 2:**
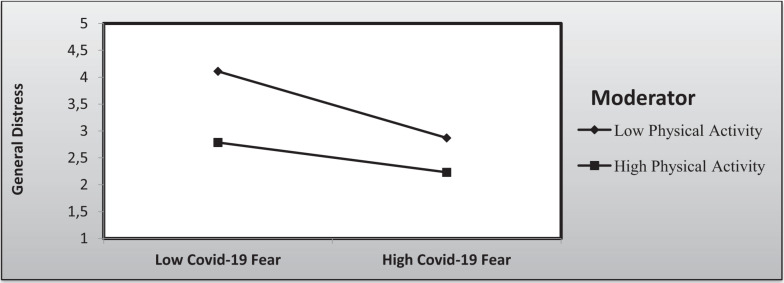
Moderation effect of physical activity on the relationship between general distress and COVID-19 fear.

Figure 2 pictorially illustrates the moderation effect of the level of physical activity on the relationship between general distress and COVID-19 fear.

## Discussion

The aim of the present investigation was to develop and assess a model that examines the impact of COVID-19 fear, level of physical activity, and gender on general distress.

Results confirmed the links between level of physical activity, fear of COVID-19, and gender. They showed a significant mediating effect of COVID-19 fear in the relationship between gender and general distress. At this level, the female gender is more afraid of COVID-19, and the mediation effect is manifested by an effect on general distress. In addition, physical activity has an effect on COVID-19, varying by gender.

Furthermore, the results highlighted the presence of a moderation effect: physical activity practitioners, even if they are afraid of COVID-19, have a lower general distress score than those who do not practice physical activity.

In line with our results, Qiu et al. (2020) attempted to measure psychological distress in the general population of China during the COVID-19 pandemic. The authors found that, with respect to men, women were more vulnerable to stress and more likely to develop post-traumatic stress disorder. Similarly, a study conducted in Iran has shown that women experience more distress. The age of the participants and their level of education did not predict distress in the two countries. In contrast, exercise hours predicted distress both in Iran and China.

Fear is an adaptive defense mechanism fundamental and instrumental to survival (Ornell et al., 2020), involving biological processes necessary to react to potentially life-threatening events. On the other hand, if chronic or disproportionate, it can become harmful and can result in various psychological/psychiatric diseases.

When individuals feel that their resources can help them cope with the demands of a given stressor, stress is perceived as a challenge, but when the demands are particularly taxing, overwhelming and straining their resources, stress becomes a threat (Blascovich, 2008). After a threat assessment, stress generally results in a series of psychological and/or physiological responses (Chrousos, 2009). The harmful effects of stress on health are well-known and include both psychological (i.e., generalized anxiety disorder, depression, post-traumatic stress disorder) and physiological (i.e., cardiovascular disease, obesity, type 2 diabetes) consequences (Thoits, 2010).

In another study, Aldana et al. (1996) examined the relationship between physical activity during leisure time and perceived stress in 32,229 adults. The results indicated that employees with high physical activity were less likely to have high perceived stress. In addition, working adults who are engaged in moderate amounts of physical activity have about half the perceived stress rate of inactive people.

Wunsch et al. (2017) have shown that physical activity and exercise during a school exam period may reduce the negative impact of stress on health outcomes. As such, sustained levels of physical activity should be maintained during periods of high stress to avoid negative effects on sleep and well-being. On the other hand, it should be noted that the relationship is reciprocal. Sloan et al. (2013) showed that a sedentary lifestyle was positively associated with an increase in psychological distress, while regular physical activity was inversely associated with it. Similar results have been reported by Hamer et al. (2010) on a large sample in Scotland and by Atkin et al. (2012) who highlighted this relationship for workers in the United Kingdom (*N* = 2,707).

However, in another study, Kim et al. (2008) examined the relationship between psychological distress and physical activity using a dose–response approach (total, professional, and leisure). A long duration (1 h/day) of physical activity was associated with a score of lower distress, but the relationship appeared to vary depending on the activity performed, with the type of activity being a determinant of the psychological benefits conferred by physical activity. Therefore, physical activity should be incorporated as part of the cognitive behavior therapy (Ho et al., 2020) and psychoeducation (Tran et al., 2020a,b) during the COVID-19 pandemic.

To summarize, physical activity appears to be an effective means of maintaining a good health status and even improving/enhancing mental and physical health during the COVID-19 outbreak. In this perspective, Jiménez-Pavón et al. (2020) have proposed physical exercise as therapy to counteract or at least mitigate the mental and physical consequences of COVID-19-induced restrictive measures especially among vulnerable groups, such as the elderly. However, the precise nature, type, and duration of this activity should be carefully evaluated and warrant further research.

### Limitations

Like all studies, the present investigation also has a number of limitations that should be acknowledged. For instance, the socio-economic status of the participants has not been explored. However, links have been highlighted between socio-economic status and COVID-19-related behaviors. Indeed Tran et al. (2020a, b) concluded that economically vulnerable populations are at the highest risk of suffering if they are affected by COVID-19. In addition, self-medication was the most widely used method to remedy health problems. Finally, the dates on which the questionnaires were filled do not coincide with the peaks of the pandemic and present some variations among countries. As such, further research is needed: a high-quality longitudinal survey could capture behavioral and psychological changes during the different phases of the outbreak.

## Conclusion and Recommendations

From the model presented in this study, the COVID-19 pandemic has an impact on psychological distress in the target populations. The effect of physical activity on psychological distress is shown to be very important during the pandemic phase. It can be recommended for stress management and adaptation during the pandemic period. In addition, many people have shown that they have not reduced their physical activities as a response to this emergency despite the stringent safety measures taken.

The results of this study suggest the following recommendations for future interventions: (1) greater attention should be paid to psychological distress which may have harmful consequences on physical and mental health, (2) physical activity can be exploited to counteract the burden of stress experienced during the containment measures implemented and enforced, (3) more attention should be paid to women to increase their level of practice of physical activity, and, (4) education and training on psychological issues should be provided to health workers and professionals in the countries under study.

## Data Availability Statement

All datasets generated for this study are included in the article/supplementary material.

## Ethics Statement

The study protocol of the present investigation received ethical clearance from the UNESCO Chair “Health Anthropology Biosphere and Healing Systems,” University of Genoa, Genoa (Italy), the College of sport sciences and physical activity, King Saud University, the Higher Institute of Sport and Physical Education of Sfax, Sfax (Tunisia), and the Faculty of Letters and Human Sciences of Sfax, Sfax (Tunisia). The Ethical Committee of the University of Sfax, Sfax, Tunisia, approved the project. The present investigation was carried out in accordance with the ethical principles of the 1964 Helsinki declaration and its subsequent amendments. The patients/participants provided their written informed consent to participate in this study.

## Author Contributions

TA, SA, DA, and NB conceived and performed the experiment. NC, NG, and FA collected and analyzed data. All authors wrote the draft and approved the final version of the manuscript.

## Conflict of Interest

The authors declare that the research was conducted in the absence of any commercial or financial relationships that could be construed as a potential conflict of interest.
